# Nursing faculty’s point of view regarding noncompliance with ethics in academic environments: a qualitative study

**DOI:** 10.1186/s12912-021-00537-y

**Published:** 2021-01-09

**Authors:** Mohsen Taghadosi, Sina Valiee, Mohammad Aghajani

**Affiliations:** 1 Trauma Nursing Research Center, Faculty of Nursing and Midwifery, Kashan University of Medical Sciences, Kashan, Iran; 2 Clinical Care Research Center, Research Institute for Health Development, Kurdistan University of Medical Sciences, Sanandaj, Iran; 3 Infectious Diseases Research Center, Faculty of Nursing and Midwifery, Kashan University of Medical Sciences, Kashan, Iran

**Keywords:** Ethics, Education, Nursing, Faculty, Students, Qualitative research

## Abstract

**Background:**

An academic environment is the first place that nursing students are introduced to ethics related to nursing and healthcare. In this study, we explored the nursing faculty members’ point of view regarding noncompliance with these academic ethics.

**Methods:**

This study was a qualitative descriptive study conducted in 2018. Faculty members at a nursing school were selected through purposeful sampling. Data was collected using semi-structured interviews. The interviews were digitally recorded and transcribed verbatim. Data collection and data analysis were conducted simultaneously. Data saturation was ensured with 11 interviews. The interview transcripts were analyzed using a qualitative content analysis method introduced by Elo and Kyngäs.

**Results:**

The participants were six women and five men with 12.72 ± 6.64 years of experience as nursing instructors. After data analysis, seven categories were identified: discrimination, violence, misuse, out-of-date instruction and knowledge, conflicts of evaluation, hypocrisy, and disorganization.

**Conclusion:**

The findings of this study indicated the existence of noncompliance regarding academic ethics. It is recommended that faculty members be informed about possible instances of ethical noncompliance in academia. There is a need to develop strategies to promote a faculty’s compliance with academic ethics. Academic administrators need to emphasize the importance of ethics in academia and use further methods to enhance academic ethics.

**Supplementary Information:**

The online version contains supplementary material available at 10.1186/s12912-021-00537-y.

## Background

Ethics are an essential and integral part in health care education and practice [1]. Just as essential, nurses are a major group of health care professionals and are at the front line of providing services for patients in health care settings [2]. They need to improve their skills to make right decisions and actions in dealing with conflicts when caring for different patients [3]. Therefore, one of the main objectives of nursing academia is to train nursing students alongside compliance with ethics in their practice [4].

Ethics in academia is a field of study that addresses a wide range of ethical issues related to academic environments [5]. Ethics in academia refers to a set of beliefs about what is assumed to be proper behavior inside and outside the classroom [6]. Faculty and educators in academic environments have key roles in developing students’ professional and ethical behaviors as well as values during their program [2, 7]. However, faculty’s role in developing nursing students’ ethical behaviors in academia is understudied [8]. There is a lack in studies on faculty’s perspectives concerning compliance with ethical behaviors in academic environments.

In a study by Aultman et al., faculty reported that establishing a proper relationship with their students is a commonly experienced ethical challenge. They explained that developing a friendly, caring relationship while maintaining a vigorous control level and ensuring productivity in classroom were among significant ethical challenges they had experienced [9]. Another research team reported that the faculty reported that there are risks of unethical incidents, such as intimate, romantic, and sexual tendencies/relationships, when building faculty-student relationships [10]. In creating an academic relationship, the faculty needs to establish an environment where the risk of dishonest behaviors is minimized, the ethical growth of students is enhanced, and ethical standards of nursing profession are valued [11]. In developing this relationship, faculty members need to be just, open, reliable, honest, sensitive about maintaining professional relationships, and respectful of uniqueness, dignity, as well as the privacy of students. Faculty members also need to avoid discriminative and autocratic attitudes towards students [12].

The role of faculty in the development of professional and ethical behaviors is significant and undeniable [13]. In general, faculty members, educators, and trainers are considered role models among students and trainees [14, 15]. The interaction between faculty members and students is one of the main factors that can influence quality and outcomes of teaching/education. Bahaziq and Crosby reported that medical students experience a wide range of unethical behaviors from medical experts and faculty during their program [16]. The unethical behaviors can negatively influence students’ educational outcomes, including learning and professional skills [17]. Therefore, a proper interaction between faculty and students is essential for improving students’ professional behaviors and their future nursing practice [18].

Compared to other faculty, nursing faculty members frequently experience more ethical conflicts and challenges as they play dual roles as a teacher, in charge of students’ in-class learning, as well as a healthcare professional/mentor [19]. In general, nursing faculty and educators have substantial roles in establishing and maintaining ethical principles amongst students, preparing the students for professional life, and monitoring the application of ethical principles in an academic environment. To advance compliance with ethical standards in academic and professional environments, it is pivotal to explore experienced, unethical behaviors based on the faculty’s point of view. Studies in the field of ethics in nursing education are mostly quantitative. However, issues relevant to ethics are multifaceted and depend on specific contexts that highlight a need for qualitative research approaches in this field. Based on faculty’s substantial roles in establishing ethical standards and behaviors in academic environments and the significance of avoiding unethical behaviors in the educational environments, the present study was conducted. The purpose of this study was to explore nursing faculty’s point of view regarding noncompliance with ethics in academic environments through a qualitative research approach.

## Method

### Design

The present study was a qualitative descriptive study conducted in 2018. This method is a systematic approach for collection, organization, and interpretation of textual materials, such as interview transcripts [20]. Qualitative content analysis was used for analyzing data and interpreting meanings in specific contexts [21]. We used a systematic coding process to analyze and interpret textual data [22].

### Participants and sampling

A purposeful sampling was used to recruit participants among faculty at School of Nursing, Kashan University of Medical Sciences, Kashan, Iran. The inclusion criteria for instructors were: having at least a master’s degree, having the tendency to participate, be willing to share experiences and perspectives, and having at least 2 years of working experience as nursing students’ instructor. A diverse sample in terms of age, gender, working experience, and academic rank was employed. The participants’ academic ranks included instructor, assistant professor, and associate professor. Sampling was continued until data saturation was ensured. Accordingly, data was collected until no additional new information was gathered from the interviews [23]. In our study, the ninth interview yielded no new data and information, which was an indicative of data saturation. After nine interviews, two further interviews were conducted to ensure comprehensiveness and saturation of data as well as to complete data collection process.

### Ethical considerations

Research approval was obtained from Kashan University of Medical Sciences Nursing School. The study was approved by the Ethics Committee of Kashan University of Medical Sciences (code: IR.KAUMS.NUHEPM.REC.1399.029). At the beginning of the study, the participants completed informed consent forms for participation in the study and the recording their interviews. Before each interview, the third author explained study objectives, reasons for recording the interviews, the voluntary nature of the study, and confidentiality of data for the participants. Interviews were conducted with the participants individually in a quiet environment and at their convenient time and place. The anonymity of participants was guaranteed.

### Data collection

Data collection was performed through semi-structured, private, and deep interviews. An interview guide was developed by researchers and used for data collection (supplementary file). The interviews began by collecting participants’ personal, clinical, and educational information. The interviews were continued by general and detailed questions concerning the participants’ experiences of faculty noncompliance with ethics in the academic environment. The question format included one main question and several detailed follow-up questions. The participants were asked to share their own experiences or those related to their faculty colleagues. The main question was: “In your point of view, what are instances of noncompliance with ethics in an academic environment and in working with colleagues and students?” To avoid possible ambiguities and to enrich the data, follow-up and detailed questions were also asked. Sample detailed question were: “Could you explain an example of experiencing noncompliance with ethics in the academic environment?” and “Why do you think this experience is an example of noncompliance with ethics?” The interviews were scheduled at a convenient place and time based on the participants’ desire. Most interviews were performed in faculty’s office at the school. The interviews lasted between 45 and 70 min and were completed by the third author. The interviews were recorded and transcribed verbatim by the third author soon after the completion of the interviews and after listening to the recorded interviews for several times. Data were collected from April to October 2019.

### Data analysis

The interview transcripts were evaluated through a qualitative content analysis method that used an inferential approach. This approach was a process of organizing qualitative data via steps of inferential content analysis; this included open coding, creating categories, and abstraction [24]. In the present study, we used a data analysis method developed by Elo and Kyngas [19]. In their study, Elo and Kyngas developed an inferential categorization method for qualitative content analysis [21]. In this method, a key point is selecting a unit of analysis from a bulk of information. Based on Elo and Kyngas, steps of analysis and categorization of data are determined based on research questions, which may be related to the whole or parts of the textual information [25]. Data collection and data analysis were conducted simultaneously. The extracted codes were categorized into primary codes based on their differences and similarities.

In the present study, units of meaning were sections of the transcript related to the research question. After determining the units of analysis, data assessment began through free-floating reading, which is reading the whole transcript several times, to achieve an overall sense and comprehension of the raw data. In the open coding stage, the transcript was read verbatim, word for word. After pondering the data, margin notes about the codes pertinent to the research questions and objectives were written. The margin notes were grouped under headings that described a specific section of the content. Primary codes were extracted from the data and those extracted codes were listed. This process was continued until categorization of the primary codes was completed [21, 26]. Coding in qualitative content analysis can be pertinent to latent content or manifested content [21, 25]. Primary coding was completed based on the participants’ words and the primary themes. Accordingly, the units of meaning were extracted from units of analysis. The units of meaning were developed based on the research question and through coding via pruning excessive units. After reading and grouping the *margin notes, headings, and primary codes,* categories emerged.

Abstraction refers to developing a general description for the subject of the study by creating categories [21]. In the present study, each category was labeled by keywords that defined the content. In this stage, subcategories were grouped into generic categories, and the generic categories were grouped into main categories.

After creating tags, the codes were categorized into subcategories. Information grouping was performed to decrease the number of categories and to help interpret the phenomenon as well as to improve in-depth understanding and insight about the data. More specialized tags were used to categorize the data. The tagged categories and subcategories were grouped into main categories or themes.

### Rigor

Overall, confirmability, credibility, dependability and transferability were established within this study to demonstrate the rigor and trustworthiness within the research [26]. The researcher spent an adequate amount of time both data gathering and participating in deep, insightful interactions with the data. The third author developed a two-way relationship with the participants, informed them about the study’s purposes, and answered their questions. Data collection and analysis were performed simultaneously. The third author reinforced the participants’ trust throughout the study’s stages, including the interviewing, continuous data collecting, voice recording, transcribing, and analyzing the data immediately after the interview, and giving feedbacks for further interviews.

To increase transferability, the author consulted with three independent faculty members who were experts in qualitative research. The collected data was presented to them for their review and feedback. The confirmability of the data was improved through the participants’ and experts’ reviews and feedback. The diversity of the participants in terms of age, gender, and work experiences was considered when selecting participants. In this step, a wide range and variety of information and data at all possible levels were included. The three authors examined the data and the process of data analysis to improve the reliability/stability of the results. Member check or respondent validation was used during the interview process and at the end of the data analysis to help improve the accuracy, credibility, validity, and transferability of the study and the results.

## Results

The participants included women (*n*= 6) and men (*n*= 5). Their age range was 34 to 59 years, and the mean age was 43.54 ± 8.45. Their mean score of working experience was 12.72 ± 6.64 ranging from 5 to 25 years. The academic rankings of the participants were instructor (*n*= 3), assistant professor (*n*= 6), and associate professor (*n*= 2). After analyzing the interview transcripts—including pruning, removing, and integrating the data—58 primary codes, 15 subcategories, and seven categories emerged. These categories include discrimination, violence, misuse, out-of-date instruction and knowledge, conflict of evaluation, hypocrisy, and disorganization.

### Discrimination

The participants reported that discrimination was an unethical behavior they frequently experienced in academic environments. Discrimination in an academic environment contained two subcategories, including *discrimination related to students* and *discrimination related to colleagues*. The participants highlighted that discrimination related to students was generally based on a student’s gender, race, or hometown. The participants also explained that due to discrimination, students frequently experienced humiliation. Humiliation can occur due to different reasons; these can include students’ course results and performance as well as their sociodemographic status, especially economic status. Furthermore, the participants reported that moments of faculty misconduct, which are mostly intentional and related to a poor student-faculty relationship, can increase a sense of humiliation among students. These misbehaviors include: hindering students’ academic achievements, depriving students an opportunity to get qualified for higher degrees or continue in their program and preventing them from achieving a decent position.

The participants believed that having a fair attitude towards students and treating them equally are substantial ethical behaviors that faculty need to consider. For instance, one of the participants stated: “Students might have different educational performances and backgrounds. Some students might demonstrate improper social behaviors. Other students might be from lower social and economic classes. The important point is that all students should be treated equally …” (P. 8).

Some participants addressed discrimination toward faculty, especially junior faculty. The participants noted that other instances of discrimination in an academic environment included connecting personal and family issues of colleagues to educational/academic environments, creating unclear task description for some senior faculty, and making unreasonable changes in some faculty’s administrational positions. A participant said: “Regardless of the faculty members’ capabilities and skills, only a few have the chance of teaching graduate-level courses. The instructors are not recognized for their capabilities even though they are highly competent and are working just like other faculty members...” (P. 6). Another participant reported this type of discrimination among colleagues in an academic environment: “Graduate-level courses are allocated to specific faculty regardless of their capabilities. Instructors only can teach available courses after assigning courses to higher-ranked faculty members. It seems that some courses specifically belong to an exclusive group of faculty members” (P. 5).

### Violence

Based on the participants’ experiences, they faced violence against two groups of individuals within an academic environment. Accordingly, the category of violence was grouped into two subcategories of *violence against students* and *violence against colleagues*. They indicated that violence is a serious issue and represents important instances of unethical and uncivil behaviors within academic environments. They believed that violence could be implicit or explicit verbal and behavioral demeanors. A participant with 3 years of experience in teaching said: “… I have seen a colleague with such a dictatorial and autocratic demeanor that students did not dare to speak to him. Students did not feel comfortable to discuss their needs and requests related to the course. Despite students’ issues with this faculty member and his behaviors, students avoid complaining and criticizing him and his course…” (P.7). Another participant stated: “…Some faculty members even commonly insult students unintentionally. The situation is worse when a faculty member says insulting or improper words to a student in front of other students. In most cases, such behaviors amongst faculty members are more common with their graduate level students…” (P. 9).

Violence against colleagues was another academic unethical behavior. A participant with 7 years of teaching experience said: “In many cases, in an academic environment, individuals misuse their colleagues’ personal and family issues to both promote or impede colleagues in their professional position. Sometimes, misusing personal and family issues are not limited to daily subjects or relationships; they are used as an excuse to replace another person with a colleague’s administrative positions…” (P. 8). Another participant said: “It is not right to spread gossip about colleagues, especially when the issues are not related to the work environment and the school at all. Such behavior would be expected among lay people not scholars” (P. 9). In a working environment, violating colleagues’ privacy, revealing their family and personal issues, and misusing these issues to abuse them are important examples of violence. Other instances of violence include using brutal or hostile behaviors and misusing own’s higher working experience to occupy or replace administrative positions. A participant with 26 years of experience mentioned: “…In some cases, faculty use forceful efforts to occupy higher administrational positions. It is like using unfair ways to win a competition, such as using doping or banned substances in competitive sports” (p. 10).

### Misuse

*Misuse* includes two subcategories: *misusing students* and *misusing colleagues*. According to the participants, misusing students by having them do faculty’s personal responsibilities or their educational and research assignments is an unethical behavior in an academic environment. One instance of this type of student misuse includes forcing students to perform faculty assignments, such as translation and data gathering for research. These activities are beyond the students’ course assignments and are not acknowledged as the students’ contributions. A participant indicated: “Instances of plagiarism including using others’ ideas and works, failing to use firsthand references, and refusing to give research funds to those who conduct the research projects are Haram (a sinful action). Moreover, there are other instances of misusing students and noncompliance with ethics in academia. For example, some faculty members refuse to acknowledge students’ contribution or including them in the list of coauthors in articles. But instead, they add other persons, who had not had any significant contributions, in articles’ list of coauthors” (P. 11). Another participant added: “Unethical research- and teaching-related behaviors are other instances of misusing students... Sometimes faculty members recruit students as samples in their research projects without students’ consent or agreement for participation” (P. 4).

The misuse of colleagues and other faculty was also reported by participants. A related unethical behavior discussed by participants was faculty’s misusing their position and office for personal benefits as well as misusing their hierarchically inferior colleagues. A participant stated: “…Assigning tasks beyond task description to a young or new colleague is a breach of regulations and laws. In many cases, senior faculty directly or indirectly compel new colleagues to do irrelevant works only for senior faculty’s personal benefits and desires” (P. 1). It is an example of misusing new faculty members be senior members.

### Out-of-date instruction and knowledge

Based on the participants’ points of view, another category of academic unethical behavior was faculty’s out-of-date knowledge and instruction. The failure to keep oneself knowledgeable and up to date regarding their career and expertise as well as methods of social interaction is considered in this category. A participant discussed: “There are rapid and ongoing advances concerning knowledge in different fields. These advances necessitate faculty members to continuously update their course material and methods. There are many related issues that cause a student’s dissatisfaction with education. For instance, students might not be satisfied with the teaching materials and methods of a faculty member because they are not revised or modified based on recent knowledge. The students discuss the issue with the faculty; however, the faculty mostly do not modify the method, and continue to teach with outdated lectures” (P. 3). Another participant stated: “…Having no prior study and preparation about the topic, merely repeating the content of the textbook, refusing to introduce references, and introducing outdated references are instances of out-of-date instruction…” (P. 2). Due to a lack of using new resources and knowledge, faculty members repeat outdated lectures and presentations for students.

### Conflict of evaluation

Conflict of evaluations in courses, including theoretical and clinical courses, was another category of unethical academic behaviors. An example surrounding conflict of evaluation includes grading students beyond any established rule such as based on relationships or the physical attractiveness/appearance of the students. One participant said: “…Whether a student wear a veil or a student’s style can influence what the student’s grade is. This is a serious and notable ethical problem” (P. 7). Considering friendship or personal relationships when evaluating an individual was also grouped in this category. A participant said: “In many cases, an evaluation of a dissertation is not related to the strengths or weaknesses of the work as well as its pros and cons. In such cases, the dissertation is evaluated only based on the student’s relationship with the advisor and whether the student has a friendly relationship with the committee members” (P. 9). This is an example of noncompliance with ethics in students’ evaluation.

### Hypocrisy

Some of the participants indicated that treating students and colleagues hypocritically was another instance of academic unethical behavior. An example is negatively talking behind another’s back in a direct or indirect way as well as one’s effort to look good in eyes of students through self-compliments. A participant said: “Sometimes, a faculty member tries to use self-compliments to convince a student that they are the only advisor who can help the student’s project goes forward. At the same time, they try to convince the student that other faculty members are incompetent in advising students” (p. 2). Another participant indicated: “There are instances of hypocrisy regarding students’ work and papers. Some faculty members lure the students and publish students’ papers/work without recognizing the students as co-authors or acknowledging their work” (p. 6).

### Disorganization

Disorganization consists of three subcategories. Subcategories of disorganization include: *disorganized theoretical education*, *disorganized presentation of educational materials*, and *disorganized clinical training*. Some participants highlighted that faculty’s failure to meet the scheduled timeframe, to follow the curriculum, to include all the educational materials in teaching, and to attend the classroom regularly and punctually are instances of unethical academic behaviors. A participant stated: “Some clinical instructors reduce the hours of students’ clinical training. For example, a clinical instructor shrinks a five-day clinical training to three days; the faculty skips two assigned days for training. In addition to poor performance and attendance, he does not use a proper assessment method for evaluating the students at the end of training” (p. 11). A participant emphasized: “An instructor is very late in clinical and then dismisses the students very early... Sometimes, he leaves the students and relegates them to others, even nurses” (P. 3). Another participant said: “One of our colleagues has no concern about the consistency between materials taught in classrooms, the curriculum, and syllabus, and he easily skips some sessions and course materials. In this regard, if the students complain, the faculty does not hear the students’ voice, and no modification is performed” (P. 1).

## Discussion

According to the participants, instances of academic unethical behaviors in a nursing school included discrimination, violence, misuse, out-of-date instruction and knowledge, conflict of evaluation, hypocrisy, and disorganization.

Discrimination refers to treating students and colleagues unequally and unjustly. As a result, educational justice is one of the principles that faculty members need to value in their teaching and professional practice. Moreover, students expect fair treatment by faculty members and academic administrators as well as an equal access to educational services. Injustice, on the other hand, creates doubts and anxiety amongst students and negatively influences students’ academic and civil behaviors [27]. Discrimination can trigger an unhealthy competition amongst students; however, when academic rules and regulations are implemented equally, the ground can be prepared for students’ healthy progress and development [28]. Respecting the principles of ethics and justice also can help promote commitment and loyalty as well as reduce the likelihood of improper and negative behaviors [27]. Educational justice can lead to satisfaction in students and has an effect on two categories of behavior amongst students: educational honesty and educational citizenship behaviors.

The concept of academic ethics covers justice as well. This concept is also associated with students’ educational commitment through mechanisms similar to justice, such as reducing doubts about trying to achieve goals [29]. A failure to observe the principles of ethics and justice in academic environments can lead students to adopt deceptive behaviors. The probability of attempting to cheat and participating in academic dishonesty is often rooted in one’s fears and concerns. Faculty members need to be cautious about behaviors, such as discrimination, that cause fears and concerns. Similarly, the faculty needs to treat students equally to reduce perceptions of injustice. In addition, academic administrators need to treat faculty members in an equal and just manner. Introducing standard measures of assessing and evaluating faculty members can help prevent faculty discrimination. Instead of focusing on faculty members’ personal issues and relationships, one needs to encourage faculty members’ high-quality performance, promote collaboration/teamwork, prevent discrimination, as well as encourage faculty members and academic administrators to improve justice and avoid discrimination.

In this present study, violence was another example of an academic unethical behavior. This refers to a situation where an individual or a group experiences potential or actual physical, mental, or spiritual threats. Violence is a type of discriminative behavior, especially when violence leads to physical and mental trauma. According to Young, violence is more than just an ethical error, it is a type of intentional dominance over others [30, 31]. Violence appears in the form of negative behaviors that threaten a person’s psychological, mental, and emotional traits. Examples of these negative behaviors include degrading one’s goals and interests, destroying hope, threatening, insulting, defaming, gossiping, bad-mouthing, humiliating, reproaching, disrespecting others, talking behind the back of others, giving sarcastic comments, and depriving individuals from opportunities [32].

Hierarchically, violence is vertical or horizontal in an academic environment. Violence amongst colleagues with the same academic rank and administrative position is an instance of horizontal violence. Horizontal violence is a wide range of non-physical, inter-group conflicts and can be visible or concealed hostile behaviors [32, 33]. Vertical violence happens both between colleagues with different academic or administrative positions as well as between faculty members and students. Fathi et al. [34] reported that according to nursing students, the main instances of vertical violence between faculty members and students were verbal violence or insulting [34]. Vertical and horizontal violence can be interconnected. For example, because of vertical violence from senior faculty members towards inferior colleagues, those inferior colleagues may redirect their negative, violent reactions toward their peers or students.

It is important to determine the types of violence and their pertinent factors in an academic environment as well as develop approaches to reduce or prevent violence in academia. Exploring violence experienced by students and faculty members can help one understand the context of violence and design preventive methods. Nursing faculty members and school administrators have a responsibility to proactively consider the likelihood and causes of violence. Accordingly, they can develop and adopt strategies to reduce the incidents of violence as well as to support people who are influenced by violence. Providing nursing students with appropriate ways to report their experienced violence is necessary. It is the educational institutions’, the faculty’s, and the academic administrators’ responsibility to design a safe method to monitor incidences of violence toward nursing students. Furthermore, it is essential to provide appropriate measures to investigate the incidences while supporting the person influenced by violence [35]. Faculty and school administrators should build empathy, convey understanding, and demonstrate a willingness to hear students’ voices to strengthen the academic community.

Misusing students was another instance of academic unethical behaviors. We considered educational ethics as the extent in which an individual’s academic behavior conforms to honesty, relies on one’s own work, avoids to use others’ work outcomes, demonstrates altruistic and morally-approved behaviors, and respects others [17]. It is essential for faculty members to centralize students’ assignments on their task description and avoid assigning tasks and assignments that are beyond the curriculum and syllabus. Extra assignments, such as student-faculty collaborations and research, need to be optional. The extra assignments need to enhance an opportunity for a student to cooperate with a faculty member and help improve the student’s skills/knowledge. Recognition of a student’s contribution by the faculty is essential. Moreover, faculty members need to employ their expertise and administrative power to help students and colleagues meet their educational and academic goals. Appropriate faculty evaluation tools are necessary to encourage students to share their concerns and recommendations through safe and perceptive methods. Using these methods can improve auditing and preventing students’ experiences of misuse and dissatisfaction in academia. It is the nursing faculty’s and administrators’ responsibility to inform students their rights and task descriptions as well as to provide confidential methods for reporting misuse and unethical academic behaviors.

According to the participants, the faculty’s out-of-date knowledge, course materials, and methods were cases of academic unethical behaviors. Rahimi et al. studied students’ points of view regarding the most important characteristics of a good instructor in four areas: research, teaching methods, communication skills, and personality. In the area of teaching, an instructor’s skills related to teaching the course as well as a provision of new and updated contents were the most important characteristics of a good instructor [36]. In another study, Gashmard, Moaetamed, and Vahedparast examined faculty members’ and students’ points of view on characteristics of a good instructor [36]. They found that the most important characteristic of a good instructor, based on faculty’s point of view, was a proper teaching method. Based on the students’ point of view, the most important characteristic was the instructor’s communication skills [37]. Rahnama et al. investigated students’ perceptions of the Student Evaluation of Instruction form as a tool for assessing instructors’ teaching effectiveness [37]. They found that the faculty members’ appropriate manners and self-esteem were the most valuable factors for effective teaching. In general, the students reported that compared to teaching and academic skills, the faculty’s skills and behaviors that were irrelevant to their academic competence were not significantly important [38]. Consistent with Rahnama et al., in the present study, the participants emphasized a greater importance concerning faculty members’ academic competence compared to their personal characteristics and personality traits.

Based on ongoing advancements of science and technology as well as subsequent changes in students’ needs and expectations, faculty members need to keep their teaching methods and knowledge updated. Innovative teaching methods require using new knowledge and avoid using old materials and methods. The sustained education of faculty as well as their continued access to the latest references, technology, and knowledge are essential for improving compliance with ethics in academia [14]. Faculty members need to enhance their knowledge and teaching methods based on the needs and expectations of their students. In their teaching methods, instructors need to engage students, use different teaching strategies, and modify the contents of the course based on students’ educational needs.

In the present study, another instance regarding noncompliance with academic ethics was a conflict of evaluation. There is a need for faculty members to consistently adhere to a standardized evaluation method for all students. In addition, the evaluation method should be clarified for students from the beginning of the course. Among available methods of student evaluation, a method that fits the course, students, and educational goals should be adopted. Additionally, a standard instructor evaluation method should be used consistently for evaluating instructors in an academic setting. Using different methods for evaluating faculty members can decrease the accuracy and strength of the evaluation. In regard to the evaluation of students, faculty members need to use strategies to reduce conflict of students’ evaluation. For example, sharing faculty experiences about students’ evaluation and discussing challenges related to evaluation can help reduce conflict of evaluation. Using methods of evaluation that promote interaction and communication between faculty members and students can be helpful in reducing conflicts of evaluation.

Hypocrisy refers to the faculty’s attention-seeking behaviors, such as making unrealistic but good impressions. We found that hypocritical behaviors can be toward students or colleagues. Honesty, however, is a person’s characteristic that does not lead to overestimating one’s capabilities and underestimating others’ capabilities. To improve ethics in academia, faculty members and academic administrators need to enhance honesty and reduce hypocrisy in their relationship with students and colleagues.

We recommend that confidentiality be valued in academic environments; faculty members need to avoid disclosing their colleagues’ private discussions and conversations. Furthermore, confidentiality of the faculty’s and students’ personal information should be observed in academic environments. Also, we recommend using measures for improving the transparency of educational processes and faculty job descriptions, recording faculty’s work activities and résumés, avoiding backbites about colleagues, and making a friendly atmosphere amongst colleagues.

Disorganization was another instance of noncompliance with academic ethics. Punctuality and discipline in a workplace are highly important in nursing profession. Faculty members are role models for students regarding professional discipline. Sarchami et al. stated that faculty violations of educational and class regulations were common among faculty members [39]. Nursing faculty members need to value discipline and punctuality in the academic environment and in clinical settings. It is necessary to observe discipline and avoid unethical behaviors in classroom. There are significant differences between clinic and classroom environments. Therefore, strategies need to be established to help faculty members organize their teaching activities and schedules in these two different settings. Establishing a reward and punishment system for encouraging professional behaviors in academia can also be considered.

In general, an academic environment is the first place that nursing students are introduced to ethics related to nursing profession. In this regard, faculty members are role models for students. It is valuable to design strategies to help nursing students improve their ethical/professional behaviors and obtain their professional identity during their nursing program. Identifying and resolving ethical issues in academic environments, especially those issues experienced by faculty members, can help prevent students’ confusion regarding ethical conflicts. Other strategies include establishing ethical codes among students, using an up-to-date knowledge base to prepare them for their professional life, and implementing ethical codes in academia, which are parts of liabilities of nursing schools. However, noncompliance with ethics amongst academic administrators and faculty are frequently reported [40]. Noncompliance with ethics in nursing education and academia may subsequently jeopardize ethics in healthcare system and clinical environments [2, 40].

### Limitations and recommendation

Noncompliance with ethics is a sensitive topic for research. Sharing perspectives and personal experiences of noncompliance with academic ethics is not a favorable topic of discussion amongst faculty members. A limitation of this study was an unwillingness of some participants to share their experiences entirely. In this study, we adopted multiple methods for improving the precision of the analysis. However, in a context-based and qualitative study, there are limitations to measure and establish reliability and precision of the study. Further studies are recommended for an exploration of a nursing faculty’s point of view on ethical issues in academic environments. Another limitation in this present study is recruiting participation from one school of nursing. Moreover, we only investigated the faculty’s points of view; there is also a need to include students’ points of view in future studies. Related seminars and workshops for faculty development can help improve professional and ethical behaviors as well as reduce ethical conflicts in academia.

## Conclusion

The participants’ point of view on their noncompliance with academic ethics were discrimination, violence, misuse, out-of-date instruction and knowledge, conflict of evaluation, hypocrisy, and disorganization. Noncompliance with academic ethics seems to be a common behavior and a wide-range phenomenon that can negatively affect nursing students’ learning and education. Nursing faculty members and academic administrators have a responsibility to identify the causes and incidents of noncompliance with academic ethics. It is necessary to design methods for nursing students to confidentially report faculty’s noncompliance with academic ethics. Furthermore, there is a need to create a safe environment for the students to discuss and share their experiences about faculty’s noncompliance with academic ethics. The results of relevant studies should be used to inform faculty about the possible instances of noncompliance with ethics and to help faculty enhance their behaviors and relationships to meet academic ethics. Moreover, there is a need to develop and advance methods for monitoring noncompliance with ethics in academic environments to increase the transparency of educational processes and reduce noncompliance with academic ethics.

## Supplementary Information

**Additional file 1.** Interview Guide.

## Data Availability

The datasets generated and analyzed during the current study are not publicly available due to their containing information that could compromise the privacy of research participants but are available from the corresponding author on reasonable request.

## Abbreviation

ICAI: International Center for Academic Integrity
