# Supplementary material for: Nursing faculty’s point of view regarding noncompliance with ethics in academic environments: a qualitative study
Source: BMC Nurs. 2021 Jan 9;20:15. doi: 10.1186/s12912-021-00537-y (PMC7797107; doi:10.1186/s12912-021-00537-y)
Supplement: Supplementary file 1 — Additional file 1. Interview Guide. [file 12912_2021_537_MOESM1_ESM.docx]

**Interview guide**:

- Introducing the researcher and the purpose of the study.
- Explaining the content of the informed consent form and answering the participants' questions.
- Signing the informed consent form and starting the voice recorder.
- Collecting participants’ personal, clinical, and educational information.
- Asking questions concerning the participants’ experiences of faculty noncompliance with ethics in the academic environment: “In your point of view, what are instances of noncompliance with ethics in an academic environment and in working with colleagues and students?”
- Asking follow-up and detailed questions: “Could you explain an example of experiencing noncompliance with ethics in the academic environment?” and “Why do you think this experience is an example of noncompliance with ethics?”
- Asking for getting more details, clarifying what the participants said, and summarizing the interview.
